# Propensity score matching for multilevel spatial data: accounting for geographic confounding in health disparity studies

**DOI:** 10.1186/s12942-021-00265-1

**Published:** 2021-02-27

**Authors:** Melanie L. Davis, Brian Neelon, Paul J. Nietert, Lane F. Burgette, Kelly J. Hunt, Andrew B. Lawson, Leonard E. Egede

**Affiliations:** 1 Ralph H. Johnson VA Medical Center, Department of Veterans Affairs, Charleston, US; 2 Department of Public Health Sciences, Medical University of South Carolina, Charleston, US; 3 The RAND Corporation, Arlington, US; 4 Center for Advancing Population Science, Medical College of Wisconsin, Milwaukee, US

**Keywords:** Average treatment effect among treated (ATT), Causal inference, Health disparities, Propensity score matching, Spatial data analysis

## Abstract

**Background:**

Diabetes is a public health burden that disproportionately affects military veterans and racial minorities. Studies of racial disparities are inherently observational, and thus may require the use of methods such as Propensity Score Analysis (PSA). While traditional PSA accounts for patient-level factors, this may not be sufficient when patients are clustered at the geographic level and thus important confounders, whether observed or unobserved, vary by geographic location.

**Methods:**

We employ a spatial propensity score matching method to account for “geographic confounding”, which occurs when the confounding factors, whether observed or unobserved, vary by geographic region. We augment the propensity score and outcome models with spatial random effects, which are assigned scaled Besag-York-Mollié priors to address spatial clustering and improve inferences by borrowing information across neighboring geographic regions. We apply this approach to a study exploring racial disparities in diabetes specialty care between non-Hispanic black and non-Hispanic white veterans. We construct multiple global estimates of the risk difference in diabetes care: a crude unadjusted estimate, an estimate based solely on patient-level matching, and an estimate that incorporates both patient and spatial information.

**Results:**

In simulation we show that in the presence of an unmeasured geographic confounder, ignoring spatial heterogeneity results in increased relative bias and mean squared error, whereas incorporating spatial random effects improves inferences. In our study of racial disparities in diabetes specialty care, the crude unadjusted estimate suggests that specialty care is more prevalent among non-Hispanic blacks, while patient-level matching indicates that it is less prevalent. Hierarchical spatial matching supports the latter conclusion, with a further increase in the magnitude of the disparity.

**Conclusions:**

These results highlight the importance of accounting for spatial heterogeneity in propensity score analysis, and suggest the need for clinical care and management strategies that are culturally sensitive and racially inclusive.

## Background

### Introduction

Type 2 diabetes is the seventh leading cause of death in the United States [1] and disproportionately affects US military veterans [2]. Not only is diabetes more prevalent among veterans [3], but veterans also experience higher comorbidity rates and increased risk of complications compared to the non-veteran population [4, 5]. The Department of Veterans Affairs has recently taken steps to address access to care through improved specialty care and emerging telehealth technologies [6]. Nevertheless, veterans continue to face a number of barriers to disease management, including wait times, geographic isolation from care facilities, and insufficient information regarding available health resources [7]. Thus, there is an ongoing need for improved disease management efforts within the Veterans Affairs (VA) healthcare system to help veterans manage their diabetes through healthy diets, regular exercise, and proper medication adherence [8].

At the same time, evidence shows that racial minorities have a higher prevalence of diabetes [9], poorer diabetes outcomes [10, 27], and higher mortality rates compared to non-Hispanic whites [11]. These disparities are explained in part by individual demographics, such as age, sex and marital status [12, 13] However, patient demographics may explain only one piece of the puzzle. Recent work examining diabetes care found that after accounting for both patient characteristics and facility-level factors, the magnitude of the disparity between non-Hispanic white and non-Hispanic black veterans in LDL cholesterol testing actually increased, with non-Hispanic blacks having lower rates of proper LDL management [14]. Studies have also shown that care providers may experience “clinical inertia”, whereby a provider fails to respond to a patient’s need for intensified treatment [15]. Indeed, a recent VA study demonstrated widespread clinical inertia in the treatment of veterans with diabetes [16]. Just as personal barriers to disease management may disproportionately affect racial minorities [17, 18], clinical inertia is also thought to be exacerbated for racial minorities whose care providers may have misleading perceptions regarding racial and ethnic minorities’ attitudes toward treatment [19]. This finding aligns with the Institute of Medicine’s position that implicit racial bias may affect clinical communication and care [20]. As a result, ongoing studies are needed to accurately quantify the extent of racial disparities in diabetes care, and to identify strategies for improved disease management.

Because racial disparity studies are inherently observational, it is necessary to account for multiple sources of confounding, both at individual and community levels, in order to obtain minimally biased estimates of race disparities. In particular, it is necessary to account not only for individual-level confounding, but also geographic confounding, which occurs when confounding factors, whether observed or unobserved, vary by geographic location. Here, we use the term “confounding” somewhat broadly to denote a general distortion of the true relationship between race and diabetes-related health outcomes [21]. Depending on the problem at hand, geographic location may act as a common cause of exposure and outcome–and hence as a true confounder–or as a mediator lying on the causal pathway between exposure and outcome. From a statistical standpoint, the two can be handled similarly, as long as the goal is to estimate the adjusted or “direct” effect of exposure on outcome. This is frequently the case in health disparities studies, as policymakers often wish to quantify the direct relationship between race and health outcomes. In the special case of geographic confounding, the goal is to appropriately account for spatial variation when estimating the extent of racial disparities.

In this paper, we seek to understand how racial minorities receive specialized care compared to a group of individuals who differ from these patients only in racial identity. Propensity score analysis (PSA) offers a principled approach to addressing this problem. Specifically, PSA enables estimation of the average treatment effect among the treated (ATT), which is of particular interest in racial disparity studies as interventions arising from these studies are typically designed to improve care for specific race groups rather than the population as a whole. Propensity score matching and weighting are two common approaches to PSA, and both can reduce bias in the estimation of the ATT. In this work, we focus on propensity score matching as it offers an intuitive approach to forming a control group that is similar to the treatment group across all factors included in the propensity score model.

While there is some previous work on propensity score matching in the context of multilevel data [22] and aggregate (region-level) data [23–25], we are interested in an integrative approach that allows spatial information to augment patient-level information through a hierarchical data structure when patients are clustered at the spatial unit. Recent work in spatial propensity score analysis for point-referenced data has focused on incorporating distance between units into the propensity score model [26]. However, this approach is not applicable to areal settings in which the spatial resolution is at a cluster level. Moreover, while this approach works well for studies of atmospheric data and environmental exposures that have broad impacts over a large spatial domain, patients in our study are clustered into discrete areal units (e.g., counties) that may share community resources that impact health more locally. We therefore extend previous work in propensity score weighting [27] and an areal-level spatial propensity score matching framework [28] that encourages matching among individuals in adjacent spatial units. While recent work suggests benefits to within-cluster matching [29], this recommendation is not easily extended to the spatial setting. In particular, spatial clusters such as counties may have very small sample sizes and may not function independently of one another in terms of policy and resources. Therefore, we utilize an approach that introduces a measure of smoothing when estimating the propensity scores for individuals in neighboring areas [28].

To this end, we augment the propensity score model with spatial random effects to account for variation due to unobserved geographic confounders. In contrast to previous work [28], the random effects are assigned scaled Besag-York-Mollié (BYM) [30, 31] prior distributions to address clustering at the spatial level and to promote localized spatial smoothing by borrowing information from surrounding geographic areas. This information sharing is critical to improving small area estimation. It also reflects our intuition that policy and community attributes may vary at the spatial level and that neighboring areas share resources and should therefore behave similarly with respect to health-related outcomes. To further differentiate this work from a prior study that found spatial PSA in addition to a spatial outcome model is superior to a spatial outcome model alone [28], we conduct a simulation study to assess the impact of ignoring geographic confounding altogether in PSA under conditions that mimic those of our application study and examine whether post-matching outcome model covariate and spatial random effect adjustment impacts the estimation of the ATT. We apply our methods to an analysis of diabetes care and education visits within the VA healthcare system. Because the VA is the largest integrated health care system in the United States, its health care decisions and policies are far-reaching; moreover, VA patients represent a “sentinel population” in health care, signaling needs of the more general public population [32]. We demonstrate that addressing geographic confounding yields improved effect estimates of racial disparities, which can in turn help guide policy decisions by motivating clinical care teams to engage patients, monitor diabetes management, and design racially and culturally sensitive strategies to alleviate disparities within the VA and beyond.

### Spatial propensity score analysis

#### Overview of propensity score matching methods

We begin by briefly reviewing the inferential properties of PSA as outlined in Rosenbaum and Rubin [33] and summarized more recently in Lunceford and Davidian [34]. Let *Z* denote a group indicator taking values 0 or 1. In theory, *Z* can represent an assigned treatment group (e.g., $Z=1$ if treated and 0 if control) or a manipulable exposure group. According to the causal framework outlined by Rubin [35], each individual is assumed to have two potential outcomes $(Y_1,Y_0)$, where $Y_1$ and $Y_0$ denote the (potentially counterfactual) outcomes under $Z=1$ and $Z=0$, respectively. The observed response, *Y*, is given by $Y=ZY_1+(1-Z)Y_0$, so that $Y=Y_1$ if $Z=1$ and $Y=Y_0$ otherwise. The causal estimand of interest depends on the clinical question at hand. Common choices are the population average treatment effect (ATE), defined as $\Delta _{ATE} = E(Y_1)-E(Y_0)$, or the population average treatment effect on the treated (ATT), defined as $\Delta _{ATT} = E(Y_1-Y_0|Z=1)$. The former provides a causal comparison between the treated and the entire control population, while the latter provides a causal comparison restricted to the treated population. The ATT is often desired in program evaluation or when the treatment is not likely to be targeted universally, as is the case in our motivating study.

As we present our work in the context of racial disparities, we acknowledge that race is not a manipulable exposure; thus, we shift our focus from inherent biological traits such as race to health professionals’ perceptions of race. As Greiner and Rubin [36] note, perceptions of race can be regarded as a “treatment” assigned to a patient at the moment of patient-clinician interaction. Here, implicit race-related biases function as the exposure, and the counterfactual outcome is the one that would be observed if such biases were eliminated. Our goal, therefore, is to create a well-balanced comparison group that is similar to the treated group across pre-treatment variables to address both patient-level and geographic confounding when estimating health disparities.

When there is no unmeasured confounding, propensity score methods can be used to derive unbiased estimators of the ATE or ATT in observational studies. The propensity score, $e(\varvec{ x})=\Pr (Z=1|\varvec{ X}=\varvec{ x})$, is the conditional probability of exposure given covariates $\varvec{ X}$, where the so-called “overlap” condition, $0<e(\varvec{ x})<1$, is assumed to hold. Propensity score matching is a technique that forms matched pairs between exposed and unexposed subjects based on the similarity of their estimated propensity scores [33, 37, 38]. As is true across all propensity score methods, matching techniques require the analyst to first decide on the form of the propensity score model (typically a logistic regression model) and the variables to be included in the model. After propensity scores have been generated, the analyst must first make decisions on the matching strategy: greedy or optimal algorithms, matching with or without replacement, the matching variable itself (e.g., propensity score or the logit of propensity score), and the rules for designating acceptable matches. Because the focus of this work is to address geographic confounding through the use of spatial random effects, our analysis strives to incorporate well evidenced propensity score methods that lend themselves to otherwise straightforward inference.

Greedy algorithms create nearest-neighbor best pair matches by iteratively choosing an individual in the treatment group, finding the control with the most similar propensity score and removing that pair from the selection process. Thus, greedy matching does not revisit matches once they are formed. Recent work has shown that greedy algorithms perform similarly to other matching procedures in their ability to form well-balanced groups [39]. Matching with replacement allows a control unit to be used in more than one pair match, whereas without replacement restricts a control to participation in only one matched pair. Matching with replacement can yield a suitable matched sample; however, a matched sample based on very few influential control units can lead to inflated variance estimates [40]. Therefore, some researchers recommend matching without replacement, which has been found to perform as well as matching with replacement but avoids analytic complexity and the variance pitfall [41]. In terms of acceptable match designation, Austin [42] recommends a caliper width equal to 0.2 times the standard deviation of the logit of the propensity score as a valuable compromise between preserving match quality and minimizing mean square error (MSE) of the treatment effect. Given the above recommendations, we adopt a nearest neighbor algorithm that matches individuals without replacement based on the logit of the propensity score and a caliper of 0.2 times the standard deviation. These choices yield a sample of treated individuals and a well-matched control group that is a subset of the entire control population, naturally allowing for estimation of the ATT. Finally, some authors recommend fitting an adjusted regression model to the outcome to address any residual imbalance between exposure groups [39], while others advocate for an unadjusted model [42]. Given this ongoing debate [43], we consider both approaches in our simulations studies to determine the preferred method in the context of spatial PSA.

#### Multi-level spatial matching

PSA has been recently extended to the hierarchical data setting in which individuals are nested within clusters such as health care plan [44]. Arpino and Mealli [22] in particular have proposed propensity score matching methods for hierarchical data that incorporate random effects into the propensity score model when within-cluster matching is not feasible. They demonstrate that random effects are capable of capturing unmeasured heterogeneity that occurs when cluster-level confounders are omitted in PSA.

The multilevel matching estimator proposed by Arpino and Mealli [22] is readily extended to the spatial setting by augmenting the propensity score model with spatial random effects. Turning to our motivating application, let $Y_{ij}$ be an indicator variable taking the value 1 if the *j*-th patient residing in the *i*-th county receives a specialty care visit, let $Z_{ij}$ be an indicator taking the value 1 if the patient is non-Hispanic black (NHB) and 0 if non-Hispanic white (NHW), and let $\varvec{ x}_{ij}$ represent a set of observed patient- and county-level covariates. The spatial propensity score model is given by

1 $$\begin{aligned} \text {logit}(e_{ij})=\text {logit}[\Pr (Z_{ij}=1|\varvec{ X}_{ij}=\varvec{ x}_{ij},\phi _{1i})] = \varvec{ x}^T_{ij}\varvec{ \beta }+\phi _{1i}, \end{aligned}$$

where $\phi _{1i}$ is the spatial random effect for county *i*. The spatial effect $\phi _{1i}$ accounts for unmeasured county-level factors associated with race, and circumvents the need to match within county, which is infeasible in the case of small cluster sizes.

Once the propensity scores are estimated, we match each NHB patient to a corresponding NHW patient to form a matched sample. The R package Matching [45] allows for direct input of the desired matching variable, is flexible enough to accommodate various strategies, and has been used in multilevel matching [29]. After matching, we follow the recommendation of Stuart [39] and fit an adjusted outcome model that can address residual imbalance across the groups with respect to important covariates and space. To fit the adjusted outcome model, we again incorporate a spatial random effect into our binary outcome model

2 $$\begin{aligned} \text {logit}[\Pr (Y_{ij}=1|Z_{ij}=z_{ij}, \varvec{ X}_{ij}=\varvec{ x}_{ij}, \phi _{2i})] = \varvec{ x}^T_{ij}\varvec{ \gamma }+ z_{ij} \alpha + \phi _{2i}, \end{aligned}$$

where $\phi _{2i}$ denotes the spatial random effect for county *i* in the outcome model. The spatial random effects can represent geographic variability in health care access, availability of community outreach and medical education programs, or access to other resources associated with diabetes management.

#### Conditional autoregressive priors

To encourage spatial smoothing in our models, we consider various conditional autoregressive (CAR) priors for the random effects $\phi _{ki}\, (k=1,2)$, where $k=1$ denote the propensity score model and $k=2$ denote the outcome model. The proper CAR prior for $\phi _{ki}$ takes the form

3 $$\begin{aligned} \phi _{ki} \mid \varvec{\phi }_{k(-i)}, \sigma ^2_k \sim \text {N} \left( \frac{\alpha }{m_i}\sum _{h \sim i} \phi _{kh} , \sigma ^2_{k}/m_i \right) , ~~k=1,2, \end{aligned}$$

where $\sigma ^2_k$ is a spatial variance component, $m_i$ denotes the number of neighbors sharing a geographic border with county *i*, $h \sim i$ indicates that county *h* is a geographic neighbor of county *i* and $\alpha$ is a spatial smoothing parameter, with $\alpha = 0$ implying spatial independence. The intrinsic conditional autoregressive (ICAR) prior [30] is a special case of the proper CAR prior in which $\alpha$ is set to its upper bound of 1, indicating maximal spatial smoothing. The resulting prior is improper and takes the conditional form

4 $$\begin{aligned} \phi _{ki} \mid \varvec{\phi }_{k(-i)}, \sigma ^2_k \sim \text {N} \left( \frac{1}{m_i} \sum _{h \sim i} \phi _{kh} , \sigma ^2_k/m_i \right) , ~~k=1,2. \end{aligned}$$

Following Brook’s Lemma [46], the joint distribution for $\varvec{\phi }_k$ = $(\phi _{k1},\ldots ,\phi _{kn})^T$ for the ICAR prior is given by

5 $$\begin{aligned} \pi (\varvec{\phi }_k \mid \sigma ^2_k) \propto \exp \left( -\frac{1}{2\sigma ^2_k} \varvec{\phi }_k^T\varvec{Q\phi }_k\right) , ~~k=1,2, \end{aligned}$$

where $\varvec{Q} = \varvec{M} - \varvec{A}$ is a spatial structure matrix of rank $n-1$, $\varvec{M}$ = diag$(m_1,\ldots ,m_n)$, and $\varvec{A}$ denotes an adjacency matrix with $a_{ii}=0$, $a_{ih} = 1$ if $i \sim h$, and $a_{ih}=0$ otherwise. When a fixed intercept is included in the model, a sum-to-zero constraint must be applied to $\varvec{\phi }_k$ to ensure an identifiable model. The ICAR model is appealing because, unlike the proper CAR, it avoids the somewhat counterintuitive assumption that the conditional mean in prior (3) is a *proportion* of the average neighboring effects. Moreover, in practice, the posterior mode of $\alpha$ tends to be close to 1, essentially resulting in a ICAR model [46].

The BYM model [30] builds upon the ICAR and adds an additional unstructured random effect to account for independent (i.e., globally rather than locally smoothed) region-level effects. Under the BYM model, the prior for $\varvec{\phi }_k$ is decomposed into the sum of an unstructured component $\mathbf {v}_{k}$ $\sim \text {N}(0,\sigma ^2_{\text {v}k} \varvec{I})$ and an independent structured component $\mathbf {u}_k$ $\sim \text {N}(0,\sigma ^2_{\text {u}k}\varvec{Q}^-)$ where $\varvec{Q}^-$ denotes the generalized inverse of $\varvec{Q}$. The resulting covariance matrix of the spatial effect is

6 $$\begin{aligned} \text {Var}(\varvec{\phi }_{k}\mid \sigma ^2_{\text {u}k},\sigma ^2_{\text {v}k})=\sigma ^2_{\text {v}k}\varvec{I}+\sigma ^2_{\text {u}k}\varvec{Q}^-. \end{aligned}$$

Thus, the BYM model allows for global shrinkage via the unstructured component, and local shrinkage vis-á-vis the spatially smoothed component. Dean et al. [47] proposed a re-parameterization of the BYM model that partitions $\varvec{\phi }_k$ into a weighted average of the unstructured and structured components:

7 $$\begin{aligned} \varvec{\phi }_k = \sigma ^2_k\left( \sqrt{1-\lambda _k}\mathbf {v}_{k} + \sqrt{\lambda _k}\mathbf {u}_{k}\right) \end{aligned}$$

with covariance matrix

8 $$\begin{aligned} \text {Var}(\varvec{\phi }_k\mid \sigma ^2_k,\lambda _k) = \sigma ^2_k\left[ (1-\lambda _{k})\mathbf {I}+\lambda _k\mathbf {Q}^{-}\right] . \end{aligned}$$

where $\lambda _{k}$ is a mixing parameter ranging between 0 (spatial independence) and 1 (ICAR). More recent work suggests that the structure matrix $\varvec{Q}$ should be scaled to ensure the the marginal variances of $\varvec{\phi }_k$ are invariant to the underlying neighborhood structure defined by $\varvec{A}$ [31, 48, 49]. Scaling unifies the interpretation of $\sigma ^2_k$ across spatial structures, thus facilitating its prior specification. The scaled BYM model takes a similar form to (7), but incorporates the scaled structured component:

9 $$\begin{aligned} \varvec{\phi }_k = \sigma ^2_k\left( \sqrt{1-\lambda _{k}}\mathbf {v}_{k} + \sqrt{\lambda _{k}}\mathbf {u}_{\star k}\right) \end{aligned}$$

with covariance matrix

10 $$\begin{aligned} \text {Var}(\varvec{\phi }_k\mid \sigma ^2_k,\lambda _{k}) = \sigma ^2_k\left[ (1-\lambda _{k})\mathbf {I}+\lambda _{k}\mathbf {Q}^{-}_{\star }\right] , \end{aligned}$$

where $\mathbf {u}_{\star k}$ is a scaled structured component and $\mathbf {Q}_{\star }$ is a scaled version of the precision matrix $\mathbf {Q}$. As in model (7), the mixing weight $\lambda _k$ is interpreted as the proportion of the marginal variance explained by the structured effect. For more information on CAR models and their variants, please see the recent review by Riebler [49].

The scaled BYM prior is appealing because it includes an unstructured spatial effect to address hierarchical clustering and a structured spatial effect to address the smooth spatial process of adjacency correlation, reflecting the intuition that patients in a county experience similar health care access, resources and environments. Moreover, by allowing for localized spatial smoothing and information sharing from surrounding geographic areas, the scaled BYM prior reduces uncertainty in estimating the propensity scores and, in turn, the ATT. Finally, by examining the estimate of $\lambda$, we can discern the degree to which the unstructured and structured components contribute to the overall variance $\varvec{\phi }_k$, and consequently, the degree to which the structures spatial component is needed. In light of these recommendations, we adopt the scaled BYM prior in the analyses below.

#### Model fitting and inference

For our case study, we adopt a Bayesian model fitting approach and assign prior distributions to all model parameters. As a default, we assign weakly informative N(0, 1e5) priors to fixed effects and Ga(1, 5e-05) priors for the spatial precision terms, where Ga(*a*, *b*) denotes a gamma distribution with shape parameter *a* and rate parameter *b*. We fit the propensity score and outcome models separately, thus avoiding the so-called “feedback” issue that can arise when the models are fit jointly under a fully Bayesian approach (McCandless et al. [53]). We use approximate Bayesian methods for posterior inference. Specifically, we adopt the efficient integrated nested Laplace approximation (INLA) proposed by Rue et al. [50]. INLA uses a Laplace approximation to estimate the joint posterior of the model parameters, yielding improved computational capabilities over standard Markov chain Monte Carlo routines. This method can be readily implemented in the R package INLA (www.r-inla.org), where the simplified Laplace approximation ([50]) is employed and the BYM2 option is used to specify the scaled BYM prior. While the advantages of the scaled BYM prior are previously mentioned, it is worth noting here that the simplified Laplace approximation corrects the Gaussian for mean and skewness and is computationally speedy, making it a solid estimation approach. The posterior means of the propensity scores are then used to match individuals.

In our application, we match individuals without replacement using the logit of the estimated propensity score with a caliper of 0.2 times the standard deviation as recommended by Austin [42]. We consider both unadjusted and adjusted outcome models when estimating the ATT. For both the unadjusted and the adjusted outcome models, we compute a “standardized” risk difference first by assuming each patient is NHB and, second, by assuming each patient is NHW. The difference provides an estimate of the ATT. In order to construct a credible interval (CrI) around this estimate, we used the inla.posterior.sample function within R-INLA to obtain 1000 Monte Carlo draws from the approximate posterior distribution. The mean of the risk difference across the 1000 samples is reported as the estimated ATT, and the corresponding the 95% CrI is derived from the 2.5 and 97.5 percentiles.

## Methods

### Simulation study

In order to assess the properties of hierarchical spatial matching, we conducted a simulation study. The goal of the study was to test the ability of spatial matching to capture the ATT and to quantify the impact of conversely ignoring space in the presence of geographic confounders, i.e. important unknown or unmeasured cluster-level covariates. Additionally, we test whether post-matching covariate adjustment improves ATT estimation.

To mirror the spatial structure of our application, we generated patient-level data clustered at the county level across the southeastern United States. To emulate the geographic structure in our application, we used the US Census county-level adjacency matrix for South Carolina, Georgia, and Alabama [51]. This matrix contains $n=272$ counties and 1528 pairwise adjacencies. We generated 100 datasets with treatment assignment and outcome according to the following propensity score and outcome models:

11 $$\begin{aligned} \text {logit}[\Pr (Z_{ij}=1|\varvec{ X}_{ij}=\varvec{ x}_{ij}, V_{i}=v_{i}, U_{i}=u_{i})]= & {} \beta _0 + \varvec{ x}^T_{ij}\varvec{ \beta }+ v_{i}\eta + f_{1}(u_{i}) \end{aligned}$$

12 $$\begin{aligned} \text {logit}[\Pr (Y_{ij} = 1|Z_{ij}=z_{ij}, \varvec{ X}_{ij}=\varvec{ x}_{ij}, V_{i}=v_{i}, U_{i}=u_{i})]= & {} \gamma _0 + \varvec{ x}^T_{ij}\varvec{ \gamma }+ z_{ij} \alpha + v_{i}\psi + f_{2}(u_{i}), \end{aligned}$$

where $i=1,\ldots , 272$, $~ j=1,\ldots ,n_i$ and $\varvec{ x}_{ij}$ is a $4\times 1$ vector comprising patient-level covariates generated from the following distributions: N (5,2), N (0,1), Bernoulli (0.4), Bernoulli (0.2). The fixed effect coefficients were set at $\beta _0$ = 0.10 and $\varvec \beta = \{ - \;0.15,0.1,0.1, - \;0.5\}$, $\gamma _{0}$ = – 1.0, $\varvec{ \gamma }= \lbrace 0.10,-0.3,0.1,-0.4 \rbrace$, and $\alpha =-0.50$. The county-level covariate $V_i$ was generated from Bernoulli (0.3) and could represent an indicator of the presence of a care facility in the region; $\eta$ was set to – 0.3 and $\psi$ was set to 0.5. The county-level covariate $U_i$ was simulated from a proper CAR model given in equation (3) with $\alpha = 0.5$ and $\sigma ^2_u={0,2,4}$ representing varying degrees of spatial variation similar to what we observe for the application study data. In order to mimic the real-life complexity with which space may enter these models, we model the relationship between this spatial covariate and exposure and outcome as a smooth function, $f_{k}(u_{i})$, which we approximate by cubic B-splines with interior knots at the first, second and third quartiles of the covariate distribution. Specifically, we let

13 $$\begin{aligned} f_{k}(u_i)=\sum ^{6}_{l=1}\nu _{kl}B_{l}(u_{i}), k=1,2 \end{aligned}$$

where $\varvec{B}_{l} = \lbrace B_{1},...,B_{6} \rbrace$ is the set of basis functions and $\varvec{\nu }_{k} = \lbrace \nu _{k1},...,\nu _{k6} \rbrace ^T$ is the vector of corresponding basis coefficients. The resulting true risk difference was $-0.10$, which is on par with our application study. Per-county sample size $n_i$ was generated uniformly within intervals defined by the quartiles of the sample sizes of our application (Min = 2; Q1 = 21; Median = 36; Q3 = 78; Max = 800). Finally, we fit models (1) and (2) using the scaled BYM prior for $\phi _k$ $(k=1,2)$. To examine the impact of ignoring spatial variation in propensity score analysis, we fit propensity score and outcome models that excluded the county-level covariates $V_i$ and $U_i$ and included only patient-level covariates.

### Analysis of racial disparities in diabetes care and management

We conducted an analysis to examine the direct association between race and the likelihood of a diabetes care visit in 2014. Our sample consisted of 20,636 NHB ($n=9,277$) and NHW ($n=11,359$) veterans with uncontrolled type 2 diabetes living in the 272 counties of Georgia, Alabama and South Carolina with a mean of 76 veterans per county (range = 2 to 800). Uncontrolled type 2 diabetes was defined as HbA1c $\ge 8$ at the start of 2014. Table 1 displays the patient-level variables that were included in the propensity score and adjusted outcome models. Approximately 13% of the patients had a diabetes care visit following indication of poor control (15.0% for NHBs, 11.2% for NHWs).

**Fig. 1:**
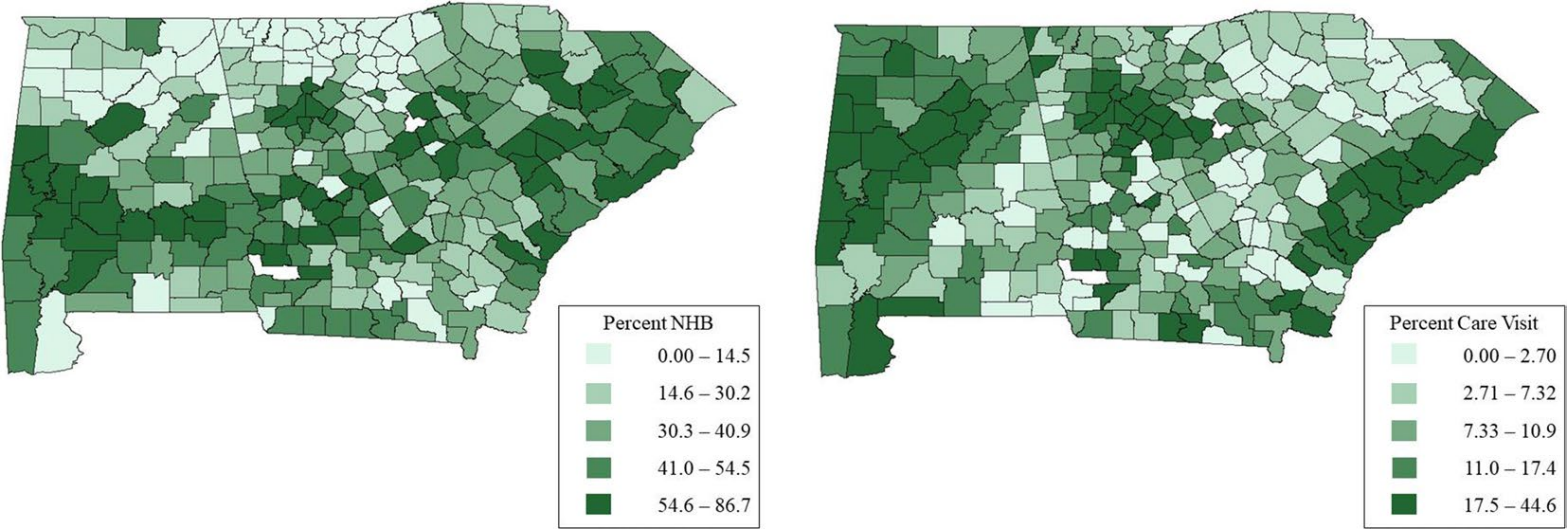
Unadjusted percent of veterans with uncontrolled diabetes who are NHB (left) and unadjusted percent of veterans with uncontrolled diabetes who received a diabetes care education visit (right)

Figure 1 displays the per-county percents of NHB veterans and veterans with diabetes care visits. The maps suggest that the percent of NHB veterans and the percent of veterans with diabetes care visits exhibit spatial variation, with clustering around areas in western Alabama, Atlanta, Georgia and coastal South Carolina.

**Fig. 2:**
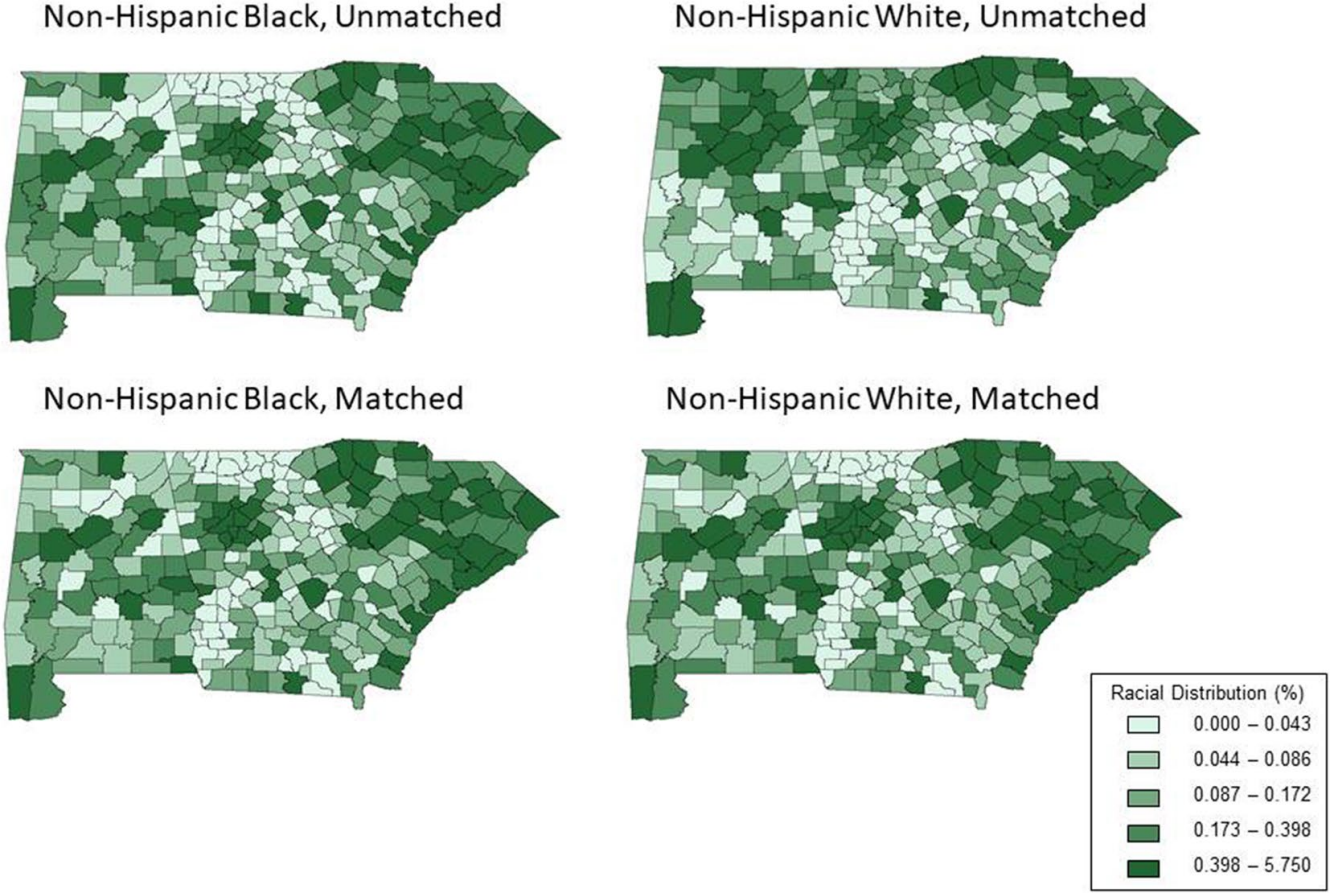
Balance of spatial distribution between NHB and NHW veterans in unmatched (top row), and spatially matched (bottom row) samples

In order to assess the covariate balance between NHB and NHW veterans in the original and spatial propensity score matched samples, we estimated the difference in means or proportions in each of the samples. To construct the spatially matched sample, we fit a logistic propensity score model that included the patient-level covariates described in Table 1 and a spatial intercept term. Matching was based on the logit of the estimated propensity score and a caliper of 0.2 times the standard deviation of the logit is imposed in order to ensure a well-matched sample. We calculated the standardized difference, a method that allows for the comparison of the relative balance of variables independent of sample size, according to Austin [42]. We observed a decrease in the standardized difference across the patient-level covariates in the spatially matched sample. However, some standardized differences were still sizable; for example, the standardized difference for “married” is close to 0.10, which would be considered the threshold for negligible difference. We were therefore motivated to consider regression adjustment in the outcome model to address any residual imbalance.

Figure 2 displays the spatial distribution of NHB and NHW veterans in the unmatched and spatially matched samples. The spatial distribution of NHB and NHW veterans varied in the unmatched sample, implying that NHBs and NHWs were concentrated in different areas. While a high percent of both NHB and NHW veterans live in urban areas such as Atlanta, NHW veterans alone appear to be concentrated in northern Georgia, where only 0.00% to 0.043% of NHB veterans reside (lightest shade on the map). This spatial imbalance is ameliorated once a spatially matched sample is created. In the spatially matched sample, the distribution of NHW veterans (the “controls”) more closely mimics the nearly unchanged distribution of NHB veterans (the “treated”), indicating that we have selected geographically well-matched controls.

To assess the performance of the proposed spatial PSA, we fit five models. We first examined the observed sample risk difference in diabetes care between NHB and NHW (“unadjusted” analysis). Next, we performed a non-spatial analysis that included patient covariates in the propensity score model, but not in the outcome model (“Patient I” analysis). Patient-level matching resulted in a sample of size 14,474. Third, we replicated the patient covariates in the propensity score model and additionally fit a covariate adjusted logistic regression model for the outcome (“Patient II” analysis). Fourth, we fit a model that included an additional spatial random effect in the propensity score model, while the outcome model was left unadjusted (“Spatial I” analysis). The spatial matching procedure resulted in a sample size of 11,110. Finally, we conducted a fully adjusted spatial PSA that included patient-level covariates and spatial random effects in both models (“Spatial II” analysis). The scaled BYM prior was used for the spatial effects in the spatial analyses. We used the estimated coefficients from the model to form a standardized estimate of the risk difference. The reported 95% CrI was constructed using the 2.5 and 97.5 percentiles of the sample distribution of risk differences.

**Table 1:** Balance of covariates between NHB and NHW veterans in pre-matched, patient-matched, and spatially-matched samples; “Stand. Diff.” denotes the absolute value of the standardized difference and “Service Percent $\ge$ 50” indicates that the veteran’s rate of service connected disability was greater than 50$\%$

Variable	Pre-matched			Patient-matched			Spatial-matched		
	NHB	NHW	Stand. Diff.	NHB	NHW	Stand. Diff.	NHB	NHW	Stand. Diff.
Age	62.00	67.49	0.573	64.26	64.23	0.003	64.51	64.54	0.003
Female	7.43	2.67	0.219	3.63	4.15	0.038	3.56	4.52	0.069
Service Percent $\ge 50$	45.97	39.41	0.133	45.15	46.61	0.041	44.09	43.80	0.008
Married	52.83	65.79	0.266	56.25	62.72	0.187	56.54	62.86	0.183
Substance Abuse	11.04	4.46	0.248	6.92	6.63	0.016	6.89	6.37	0.030
Cerebrovascular Disease	3.58	3.24	0.019	3.70	3.57	0.010	3.91	3.19	0.055
Congestive Heart Failure	8.58	11.07	0.084	9.30	9.42	0.006	9.97	8.84	0.055
Cardiovascular Disease	8.46	15.64	0.222	9.96	10.03	0.003	11.00	9.95	0.048
Depression	35.94	31.55	0.093	34.78	35.46	0.020	34.37	33.84	0.016
Hypertension	88.41	84.44	0.116	87.62	87.03	0.025	87.25	86.43	0.034
Obesity	27.23	25.87	0.031	27.04	26.89	0.005	27.04	26.59	0.014
Psychoses	6.83	3.81	0.135	5.44	4.93	0.033	5.47	4.95	0.033
Homeless	0.91	0.18	0.099	0.75	0.25	0.100	0.70	0.25	0.093

**Table 2:** Simulation Study Results: Relative bias (Bias), RMSE, and 95% coverage (Cov.) of the risk difference under various spatial variances (rows) and estimation methods (columns)

	Non-spatial						Spatial					
	Unadjusted			Adjusted			Unadjusted			Adjusted		
	Bias	RMSE	Cov.	Bias	RMSE	Cov.	Bias	RMSE	Cov.	Bias	RMSE	Cov.
$\sigma ^2_{u}=0$	0.043	0.005	96	0.040	0.005	95	0.035	0.004	95	0.050	0.006	94
$\sigma ^2_{u}=2$	0.084	0.011	77	0.085	0.011	75	0.064	0.009	90	0.052	0.007	92
$\sigma ^2_{u}=4$	0.096	0.012	70	0.100	0.012	66	0.064	0.008	93	0.049	0.006	95

$\sigma ^2_{u}=0$ represents a non-spatial scenario that excludes county-level covariates $V_{i}$ and $U_{i}$. $\sigma ^2_{u}=2$ and $\sigma ^2_{u}=4$ represent spatial scenarios that include county-level covariates $V_{i}$ and $U_{i}$ where the spatial variance of $U_{i}$ is either 2 or 4, respectively

**Table 3:** Estimated risk differences in the racial disparity of diabetic care visits under modeling strategies

Model	Risk difference	95% CrI
Unadjusted	0.038	(0.029, 0.047)
Patient I	– 0.021	(– 0.031, – 0.011)
Patient II	– 0.027	(– 0.038, – 0.016)
Spatial I	– 0.057	(– 0.068, – 0.046)
Spatial II	– 0.073	(– 0.091, – 0.056)

Negative values indicate that NHBs have a lower estimated risk of receiving a diabetic care visit. Unadjusted: observed sample risk difference. Patient I: covariate-adjusted propensity model, unadjusted outcome model. Patient II: covariate-adjusted propensity score and outcome models. Spatial I: spatial propensity score model, unadjusted outcome model. Spatial II: spatial propensity score and outcome models

## Results

### Simulation study

Table 2 summarizes the results of the first simulation study. This table presents measures of performance of spatial propensity score matching under varying degrees of spatial variation. Rows indicate the spatial variance values; columns indicate whether spatial random effects were incorporated in the analysis. Within each strategy, columns further indicate whether the estimate was derived from an unadjusted model that included an indicator for race only or an adjusted model that included patient covariates with and without an additional spatial random effect. Explicitly, “Non-Spatial, Unadjusted” implies that the propensity score model included only individual-level covariates, while the outcome model included only a indicator for race; “Spatial, Unadjusted” implies that the propensity score model included both individual-level covariates and a spatial random effect, while the outcome model included only race; “Non-spatial, Adjusted” implies that the propensity score and outcome models ignored space but included individual-level covariates; and “Spatial, Adjusted” implies that propensity score and outcome models included both individual-level covariates and a spatial random effect.

Several trends are apparent in Table 2. First, ignoring geographic confounding and utilizing only patient-level measures is detrimental. We observe poor performance of the non-spatial analyses as the bias and RMSE are increased while the coverage is decreased. Secondly, regression adjustment appears to yield smaller bias and RMSE than unadjusted analysis and typically better coverage when spatial analyses are performed. For example, when $\sigma ^2_{u}=2$ and space is ignored, coverage is 77% and 75% in the unadjusted and adjusted analyses, respectively. However, when spatial PSA is conducted for the same data, we observe reasonable coverage, with the additional adjustment in the outcome model yielding a slightly better result than the unadjusted outcome analysis (90% versus 92%). The results are even more notable for $\sigma ^2_{u}=4$. Lastly, in the case of no true spatial heterogeneity, conducting spatial analysis does not appear to be highly detrimental, as it contributes no additional bias. For instance, when $\sigma ^2_{u}=0$ and county-level covariates $V_{i}$ and $U_i$ are excluded from the data generating models, non-spatial and spatial analyses yielded nearly identical coverage probabilities. We observe similar trends in measures of bias and RMSE. These results suggest that incorporating spatial random effects into the propensity score model and the adjusted outcome model yields favorable results when unmeasured geographic confounding is present and does not yield negative consequences when the data exhibit no spatial heterogeneity.

### Analysis of racial disparities in diabetes care and management

Results of this stepwise analysis are presented in Table 3. The unadjusted risk difference indicates that NHB veterans with uncontrolled diabetes have a greater probability of receiving diabetes care and education (risk difference = 0.038, 95% CrI = [0.029, 0.047]). This result is somewhat counterintuitive in comparison to other studies on care management, which have found that NHBs are less likely to receive intensified treatment [19, 52]. However, NHB veterans included in our study were more likely to be obese, female, and have a higher rate of service connected disability (Table 3). The imbalance in these factors may explain the positive direction of the disparity. Once we matched on patient level factors (“Patient I”), the risk difference reversed direction (– 0.021, 95% CrI = [– 0.031, – 0.011]), indicating that NHB veterans with uncontrolled diabetes have a lower probability of receiving specialized care and education. With further covariate adjustment in the outcome model (“Patient II”), the risk difference decreased slightly (– 0.027, 95% CrI = [– 0.038, – 0.016]) but was similar to the estimate from the matched sample unadjusted model. Because the percent of NHB veterans and the percent of veterans receiving care visits by county appear to exhibit spatial variation, it is likely that when geography is ignored, the true disparity is not fully revealed, as NHBs may be more likely to live in areas with high rates of care visits. When spatial random intercepts were included in the analysis and the matched sample was geographically balanced, we observed an increase in the magnitude of the disparity. In the unadjusted spatial analysis (“Spatial I”), the estimated risk difference was – 0.057 (95% CrI = [– 0.068, – 0.046]). In the adjusted spatial analysis (“Spatial II”), the estimated risk difference was – 0.073 (95% CrI = [– 0.091, – 0.056]), suggesting a 7 percentage point difference in the receipt of diabetes care between NHBs and NHWs. While in general agreement with the effect estimate from the unadjusted spatial analysis in the matched sample, the effect estimate from further regression adjustment indicates a more marked racial disparity, providing strong evidence for the incorporation of spatial random effects in both the propensity score and outcome models. Lastly, results indicate that a high proportion of the marginal variance was explained by the structured spatial effect, with $\hat{\lambda } = 0.89$ and 0.92, respectively, for the propensity score and adjusted outcome models in the Spatial II analysis.

## Discussion

We have tested and employed a two-stage spatial propensity score matching framework in comparison to a one-stage and non-spatial framework to estimate the ATT among racial groups in studies examining disparities in health management and system engagement. To account for unmeasured geographic confounding, we incorporated spatial random effects into the propensity score model and, in the case of further regression adjustment, into the outcome model as well. These spatial effects can represent geographic confounders such as proximity to health care facility, access to resources, and community support and education. The spatial effects were assigned scaled BYM priors that address clustering and promote local spatial smoothing and are able to improve estimation in areas with sparse data. We adopted a Bayesian inferential approach, but fit the propensity score and outcome models separately to avoid potential feedback concerns that arise from joint estimation [53]. By implementing Bayesian estimation within R-INLA, we used readily available, free software that can be utilized in a multitude of studies across many health care data platforms.

In simulation, we examined the performance of the spatial propensity score matching framework under varying degrees of spatial variation. Under true geographic confounding, spatial matching outperformed matching that failed to incorporate spatial information. Spatial matching demonstrated decreased bias and RMSE and improved coverage compared to non-spatial matching. This result was true whether the ATT was estimated by unadjusted regression in the matched sample or further covariate and spatial adjustment was employed. In general, regression adjustment to address residual imbalance led to lower bias and RMSE. When true geographic confounders were ignored in the analysis, and only a spatial random intercept was included in the modeling, spatial matching offered reasonably low bias and RMSE and nearly nominal coverage, suggesting that the proposed method can alleviate bias due to omitted spatially varying confounders. In contrast, the non-spatial analysis performed very poorly. This supports the need to address geographic confounding in studies of racial disparities.

Our application explored the impact of geographic confounding in racial disparities among veterans with uncontrolled diabetes in the southeastern United States. We reported an unadjusted estimate of the ATT, a patient-level matched estimate, a spatially matched estimate, and a spatially matched estimate that further addressed imbalance through an adjusted regression model. The crude unadjusted estimate suggested that NHB veterans with uncontrolled diabetes may have a higher probability of receiving a specialty care visit; however, once patient-level factors were balanced, the estimate suggested that these NHB veterans may actually be less likely to receive specialty care. Furthermore, once we additionally balanced on space, the disparity in diabetes care visits became more pronounced, with NHB veterans having a lower probability of receiving a specialty care visit to address their uncontrolled diabetes.

These findings contribute to the body of literature that characterizes racial differences in the receipt of care for vulnerable patients. This cumulative body has important policy implications for mitigating disparities in diabetes management and for improving patient engagement with the health care system. First, policymakers can target intervention to identify, engage and maintain patients who are in need of intensified treatment. Vulnerable populations who are less likely to seek specialized care may need to be recruited in local, well-trusted community settings [54–56]. These patients may also benefit from care navigators or patient advocates in a complex care setting [57]. Clinician training can be tailored to address issues such as implicit racial biases resulting in “clinical inertia” and the conduct of culturally sensitive consultations [58]. Lastly, disease management media and instruction pamphlets can encourage patients to seek guidance and agency of their clinical care. These policy efforts can help the VA achieve its stated mission to “champion advancement of health equity and reduction of health disparities for disadvantaged veterans” as outlined in its recent Health Equity Action Plan [59].

Future work could adapt spatial propensity score methodology to stratification or a combination of propensity score methods. Furthermore, the proposed methods could be extended to accommodate time-varying treatments or broader types of outcomes, such as count, survival or multivariate outcomes. Lastly, the work presented here could be applied to numerous other public health applications, such as studies addressing the implementation of telemedicine or spatially varying outreach programs.

## Conclusions

The results of this work emphasize the importance of accounting for spatial heterogeneity in propensity score analysis in the presence of geographic confounding. Geographic confounding, when present, can be addressed through the inclusion of spatial random effects in a two-stage propensity score analysis framework. When geographic confounding is not present, this approach is unlikely to induce any detriment in effect estimation but may require additional computing resources. The results of our racial disparities study in diabetes specialty care suggest the need for clinical care and management strategies that are culturally sensitive and racially inclusive to address the potential for implicit bias and clinical inertia.

## Data Availability

Data used in this study is not publicly available and must be requested through the Department of Veterans Affairs.
